# Soft tissue and water substitutes for megavoltage photon beams: An EGSnrc‐based evaluation

**DOI:** 10.1120/jacmp.v17i1.5700

**Published:** 2016-01-08

**Authors:** Ambreen Aslam, Muhammad Basim Kakakhel, Shaukat Ali Shahid, Lubna Younas, Sobia Zareen

**Affiliations:** ^1^ Department of Physics & Applied Mathematics Pakistan Institute of Engineering & Applied Sciences (PIEAS) Nilore Islamabad Pakistan; ^2^ Departement of Physics University of Agriculture Faisalabad Pakistan

**Keywords:** Monte Carlo, tissue substitutes, PMMA, polystyrene, EGSnrc, PDDs

## Abstract

In this work, soft‐tissue equivalence of water, polystyrene, PMMA and water equivalence of polystyrene, and PMMA has been assessed for multiple megavoltage photon beams and field sizes. EGSnrc based Monte Carlo (MC) codes, BEAMnrc and DOSXYZnrc are used for the linac head modeling and the phantom dose calculations, respectively. Percentage depth doses (PDDs) are scored for two field sizes ($5\times 5\;{\text{cm}}^{2}$, $10\times 10\;{\text{cm}}^{2}$) and photon energies (6 MV and 10 MV) in water, polystyrene, PMMA, and soft tissue. The comparisons of PDDs show that soft‐tissue equivalence of various materials varies with the depth in the phantom, field size, and photon energy. Water and PMMA are found to be the closest soft‐tissue and water substitutes, respectively. Soft‐tissue and water equivalence of dosimetry materials need to be evaluated for a range of photon energies and field sizes before their application in complex radiation beams.

PACS numbers: 87.55.Gh, 87.55.K‐

## I. INTRODUCTION

Global cancer burden stood around 14 million new cases in 2012, and is projected to 22 million in the next two decades.^(1)^ Radiation therapy or radiotherapy is a cost‐effective cancer curative modality. However, to achieve a higher tumor control probability (TCP) and lower normal tissue complication probability (NCP), accurate radiotherapy dose calculations are an essential requirement.^(2)^ A number of radiotherapy dose estimation algorithms are available,^(3)^ including random number‐based Monte Carlo (MC) techniques.^(4)^ MC methods exhibit superior accuracy in modeling scatter and dose perturbations, especially at nonhomogeneous interfaces.^(5)^ Commercial MC treatments planning systems (TPS) are now available in the clinic.^(6)^ Besides these MC‐TPS, many general purpose radiation transport packages, such as EGSnrc, PENELOPE, MCNP, and GEANT4, are commonly employed for radiotherapy dose evaluations.^7^, ^8^, ^9^, ^10^ Amongst these, EGSnrc is widely utilized and has been benchmarked.^(11)^ This code simulates coupled electron–photon transport and also provides a number of subcodes — for instance, BEAMnrc^(12)^ used for modeling linac head and DOSXYZnrc^(13)^ employed for patient/phantom dose scoring.

Water is a standard choice for radiotherapy dosimetry. It is also considered to be a closer soft‐tissue–equivalent material because of its comparable effective Z, mass attenuation, and absorption coefficients. However, in routine clinical use many soft‐tissue/water‐equivalent dosimetry materials, such as polymethyl methacrylate (PMMA), polystyrene RW3, VW, and PAGAT, provide a more flexible dosimetry solution.^(14)^ The use of water‐equivalent materials for electron dosimetry and their soft‐tissue equivalence for diagnostic radiology has been reported.^14^, ^15^, ^16^ Still, in the case of megavoltage photon beams such data for various materials, field sizes, and photon energies are limited. EGSnrc provides a flexible solution for such equivalence studies, where a number of input parameters (e.g., field size, incident beam energy, phantom material & dimensions, and voxel sizes) can be changed, and relevant dosimetric quantities can be retrieved. EGSnrc default material list includes limited number of water/soft‐tissue–equivalent materials such as PMMA and polystyrene. However, to model other materials like RW3 and PAGAT, the cross‐sectional data need to be generated in EGSnrc. Therefore, the present study is restricted to EGSnrc default materials only.

This study utilizing EGSnrc aims to compare: a) water, PMMA, and polystyrene for their soft‐tissue equivalence, and b) PMMA and polystyrene for their water equivalence, at multiple photon energies and field sizes.

## II. MATERIALS AND METHODS

The MC simulations were carried out in two steps. First, linac head modeling with BEAMnrc and, secondly, phantom dose calculations in DOSXYZnrc for various soft‐tissue– and water‐equivalent materials. The linac head of a Varian Clinac 2100 (Varian Medical Systems, Palo Alto, CA) was simulated according to the vendor's specification as reported earlier.^(17)^ The incident electron beam was modeled as an elliptical Gaussian profile with 0.1 cm width (source 19 in BEAMnrc). To increase the efficiency of simulations, directional Bremsstrahlung splitting (DBS) was used with splitting number of 1000 and splitting field radius of 5 cm and 10 cm for $5\times 5\;{\text{cm}}^{2}$ and $10\times 10\;{\text{cm}}^{2}$ field sizes, respectively. Global ECUT and PCUT values were chosen to be 0.7 MeV and 0.01 MeV, respectively. Phase space files were generated at 55 cm from the target for 6 MV and 10 MV photons with $5\times 5\;{\text{cm}}^{2}$ and $10\times 10\;{\text{cm}}^{2}$ field sizes.

For phantom dose calculations, the previously generated phase space files were used as an input to DOSXYZnrc (source 2 full phase space source file) for scoring percentage depth dose curves (PDDs) in a virtual phantom with dimensions of $15\times 15\times 45\;{\text{cm}}^{3}$. The selected phantom size ensured the adequate modeling of the lateral scatter for the two field sizes without incurring extra computational burden. DBS values identical to those in the BEAMnrc were used. Phantom materials included the EGSNRC default: ICRUTISSUE700ICRU, PMMA700ICRU, POLYESTY700ICRU and H2O700ICRU. In Table 1, material properties, such as effective atomic number, electronic density, mass attenuation coefficient, and mass energy absorption coefficient, are listed.^(18)^

**Table 1:** Material properties.^(18)^

*Phantom Material*	$Z_{\text{eff}}$	*Mass Attenuation Coefficient* ($m^{2}/\text{kg}$)	*Mass Energy Absorption Coefficient* ($m^{2}/\text{kg}$)	*Electron Density (e/g)*
Soft Tissue	7.64	$2.74\times 10^{-3}$ (6 MV)	$1.78\times 10^{-3}$ (6 MV)	$3.32\times 10^{23}$
		$2.19\times 10^{-3}$ (10 MV)	$1.54\times 10^{-3}$ (10 MV)	
Polystyrene	5.74	$2.62\times 10^{-3}$ (6 MV)	$1.70\times 10^{-3}$ (6 MV)	$3.23\times 10^{23}$
		$2.06\times 10^{-3}$ (10 MV)	$1.44\times 10^{-3}$ (10 MV)	
PMMA	6.56	$2.65\times 10^{-3}$ (6 MV)	$1.72\times 10^{-3}$ (6 MV)	$3.24\times 10^{23}$
		$2.105\times 10^{-3}$ (10 MV)	$1.48\times 10^{-3}$ (10 MV)	
Water	7.51	$2.77\times 10^{-3}$ (6 MV)	$1.80\times 10^{-3}$ (6 MV)	$3.34\times 10^{23}$
		$2.219\times 10^{-3}$ (10 MV)	$1.56\times 10^{-3}$ (10 MV)	

Voxel sizes in X and Z dimensions were set as $0.5\;{\text{cm}}^{3}$, whereas in Y direction only three voxels (14.5 cm, 0.5 cm, and 14.5 cm) were defined to reduce the computational overhead. The PDDs were intercompared using percentage difference method. The DOSXYZnrc simulations were run with 1.5 billion initial histories to achieve a statistical uncertainty of less than 1% in MC results. The uncertainty in the methods includes the inherent approximation in the electron‐photon transport and the cross‐sectional data libraries of the MC code (physics model), the voxel size, and the number of histories both determining the overall statistical error. However, these are systematically present in the all the simulations (i.e., for the reference material and the materials being compared). Therefore, the relative results will not be greatly affected. Simulations for both phases were run on an Intel Core I3 machine (Intel Corporation, Santa Clara, CA) with 4 GB RAM.

## III. RESULTS

### A. Soft‐tissue equivalence of PMMA, polystyrene, and water

**Figure 1:**
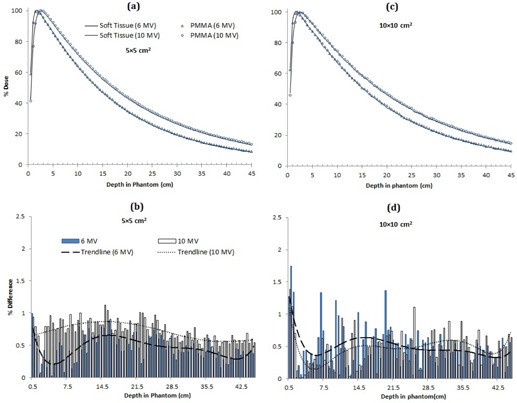
Comparisons of PDDs and percentage differences of PMMA and soft tissue at 6 MV and 10 MV energies for field size $5\times 5\;{\text{cm}}^{2}$ ((a) and (b)) and $10\times 10\;{\text{cm}}^{2}$ ((c) and (d)).

In Fig. 1(a) to (d) the PDD and percentage difference comparison of PMMA and soft‐tissue phantoms are presented for 6 MV and 10 MV photons at $5\times 5\;{\text{cm}}^{2}$ and $10\times 10\;{\text{cm}}^{2}$ field sizes. A 6th degree polynomial is fitted into the pixel‐by‐pixel data to illustrate the percentage difference trend in Fig. 1(b) and (d), and subsequently for the similar comparisons in 2, 3.

From Fig. 1(a) and (c) it is evident that, for both the energies, the PMMA–soft‐tissue PDDs are in better qualitatively agreement for the $5\times 5\;{\text{cm}}^{2}$ field size. This is also confirmed by the larger percentage differences in Fig. 1(d) for $10\times 10\;{\text{cm}}^{2}$, where the maximum difference is greater than 1.5%. In the case of smaller field size of $5\times 5\;{\text{cm}}^{2}$ in Fig. 1(b), the maximum difference is slightly above 1%. For $10\times 10\;{\text{cm}}^{2}$, the percentage differences in the first half of the phantom are greater for 6 MV, while in the second half these are higher for the 10 MV beam (Fig. 1(d)).

**Figure 2:**
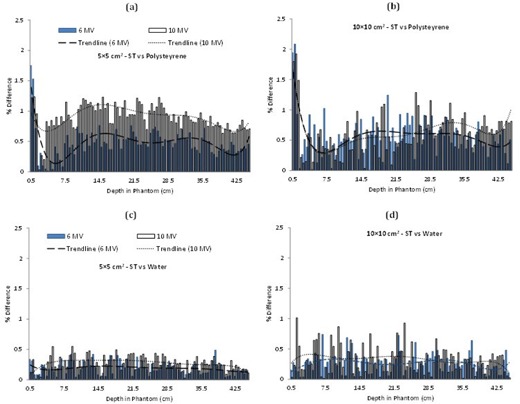
Comparisons of percentage differences at different energies and field sizes: ((a) and (b)) polystyrene–soft tissue and water–soft tissue ((c) and (d)).

**Figure 3:**
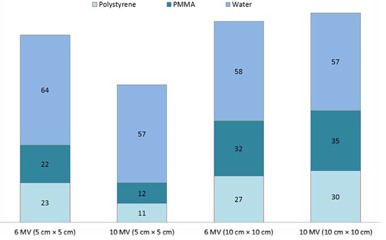
Number of voxels in PMMA, polystyrene, and water phantoms having percentage error less than 1%.

Figure 2 assesses the polystyrene–soft tissue (Fig. 2(a) and (b)) and the water–soft tissue (Fig. 2(c) and (d)) scenarios, respectively. PDDs similar to those reported in Fig. 1 are scored but not shown here. In Fig. 2, polystyrene and water exhibit similar percentage difference trend, as reported for PMMA in Fig. 1(c) and (d). However, value of the percentage difference for soft tissue–water (Fig. 1(c) and (d)) has dropped for both the field sizes and photon energies as compared to the polystyrene–PMMA case.

Figure 3 indicates the number of voxels for each material having less than 1% error (as compared to soft tissue). Here, water has the highest number of voxels passing this criterion for both field sizes and photon energies, while PMMA is the next, and last is polystyrene.

Table 2 presents the mean percentage errors for soft tissue and water equivalence. To avoid the buildup fluctuations, calculations are done in the postbuildup region for each material. For the soft‐tissue equivalence, the error in water is the least, although this is dependent on the energy and the field size. For the 6 MV photons, the smaller field size is showing less error, whereas, for the 10 MV beam, the larger field size is showing less error, except for the case of water. The use of higher photon energy results in the increased mean percentage errors for the both field sizes.

**Table 2:** Mean percent error for soft‐tissue and water equivalence.

*Error Test*	*Energy Field Size*	$6\;\text{MV}\;5\times 5\;{\text{cm}}^{2}$	$6\;\text{MV}\;10\times 10\;{\text{cm}}^{2}$	$10\;\text{MV}\;5\times 5\;{\text{cm}}^{2}$	$10\;\text{MV}\;10\times 10\;{\text{cm}}^{2}$
Soft‐Tissue Equivalence	PMMA	1.02	1.13	1.17	1.38
	Water	0.50	0.86	0.57	0.74
	Polystyrene	1.21	1.4	1.49	1.80
Water Equivalence	PMMA	1.32	1.57	1.63	1.77
	Polystyrene	1.57	1.92	1.97	2.24

### B. Water equivalence of polystyrene and PMMA

**Figure 4:**
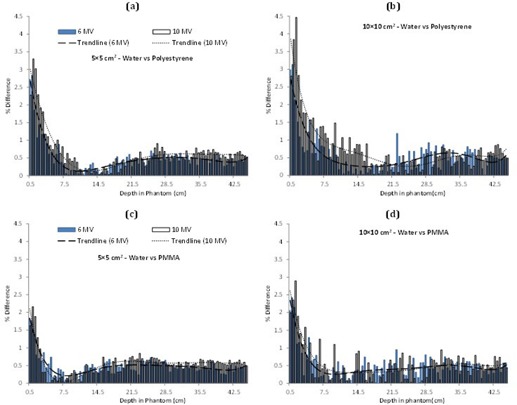
Comparisons of percentage differences at different energies and field sizes: water and polystyrene ((a) and (b)) and water and PMMA ((c) and (d)).

In Fig. 4, the percentage differences of polystyrene (Fig. 4(a) and (b)) and PMMA (Fig. 4(c) and (d)) with respect to water are shown. The difference has increased with the increase in the energy from 6 MV to 10 MV for both the materials and the field sizes. However, PMMA shows less deviation from the water behavior as compared to polystyrene.

**Figure 5:**
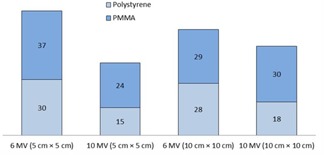
Number of voxels in PMMA and polystyrene phantoms having percentage error less than 1%.

Figure 5 shows the number of voxels of polystyrene and PMMA having less than 1% error (as compared to water). For both the field sizes and the photon energies, PMMA results are superior than polystyrene. Mean percentage errors for PMMA are less than 2% (Table 2). At the same time, polystyrene has higher percentage errors and its mean percentage error is greater than 2% for 10 MV beam for the $10\times 10\;{\text{cm}}^{2}$ field size. These results indicate that PMMA is a better water substitute compared to polystyrene.

## IV. DISCUSSION

Soft‐tissue equivalence of water, PMMA, and polystyrene, and water equivalence of PMMA and polystyrene is evaluated for 6 MV and 10 MV photons for $5\times 5\;{\text{cm}}^{2}$ and $10\times 10\;{\text{cm}}^{2}$ field sizes. The results in 1, 3 show some general trends, such as higher error in the buildup region for both the energies and the field sizes. This can be attributed to the transient region where the secondary electrons have not deposited their full energy. It also suggests that the materials being investigated for equivalence are not adequately replicating the dose deposition pattern in this part of the phantom. Furthermore, the percentage difference generally starts with a higher value and then decreases with the increase in the phantom depth as the transient equilibrium is established. However, further increase in the depth again increases the percentage difference. This can possibly be attributed to the increased scatter with the depth in the phantom. Moreover, in some cases towards the end of the phantom, the percentage difference starts to increase again, a phenomenon likely linked to the disruption of the lateral scatter equilibrium at the phantom boundary.

In 1, 3, for smaller field size the percentage difference trend lines for 6 and 10 MV do not cross over. However for larger field size, single crossovers are visible for soft tissue–PMMA and soft tissue–polystyrenes cases (1, 2), while water shows multiple crossovers (Fig. 2(d)). This is because of the increased dose discrepancy in the large field size, a possible outcome of the increased scatter. These dose deviations are driving the line fitting parameters, thus possibly resulting in the crossovers.

The photon interactions involved for these energy ranges are mostly (about 80%) Compton interactions, with a rare chance of pair production (about 20%). The Compton interactions are independent of the effective atomic number of the phantoms, and for this reason the dose absorption rates for any material should be independent of its Z. Pair production, on the other hand, depends on Z^2^ of the phantom materials. The results for soft‐tissue equivalence indicate water to be a better soft‐tissue substitute. For a material to be soft‐tissue equivalent its electronic density, mass attenuation coefficient, mass energy absorption coefficient, atomic number of the constituent elements, and their fraction by weight, should be same or close to that of soft tissue. Soft tissue and water have the same electronic densities; there is only 0.1 eV difference in the mean excitation energies of water and soft tissue. Closer mass attenuation coefficients, mass energy absorption coefficient, and fraction by weight for same atomic number (as given in Table 1) also make water a better soft‐tissue substitute. After water, PMMA indicates greater soft‐tissue equivalence as compared to polystyrene, as evident from the results in Fig. 2 and Table 2. Field size and energy dependency of the results in 2, 4 can be seen in the light of scatter and mode of gamma ray interaction. The mean percentage errors in Table 2 show that increasing the field size has amplified the error for all the materials. Larger field size produces more collimator scatter, which also is a function of photon energy and phantom material. The change in the percentage difference with photon energy is a function of increased pair production probability, which is related to the effective Z of a material.

Evaluation of water equivalence of PMMA and polystyrene indicates that PMMA is a more water‐equivalent material. This is expected due to its 32% oxygen component as compared to water having 89% oxygen. These results confirm the previous findings by Palm et al.^(19)^ whereby PMMA, because of its oxygen constituent, is shown to be more water‐equivalent than polystyrene. For polystyrene, which is entirely composed of hydrogen and carbon, the percentage difference is greater as compared to PMMA (Table 2). Doses for water and its candidate substitutes in the buildup regions differ more than in the postbuildup regions. It would be interesting to revisit the previous water‐equivalence results of Thwaites^(16)^ and Borcia and Mihailescu^(15)^ for electron beams. Thwaites tested clear polystyrene and solid water using ion chamber measurements, whereas Borcia evaluated WT1, PMMA, and polystyrene for their water equivalence in electron dosimetry using MC techniques. The results of Thwaites showed that solid water is a better water substitute as compared to clear polystyrene for electron dosimetry; however, its results are still different from that of water. Borcia and Mihailescu concluded PMMA and WT1 (Solid Water) to be water‐equivalent as compared to polystyrene. Both these studies did not consider the soft‐tissue equivalence problem for megavoltage photon beams which has been addressed in the current study.

As has been demonstrated above, the soft‐tissue equivalence of PMMA, polystyrene, and water indicate that water is a better soft‐tissue substitute, followed by PMMA and polystyrene. In the case of water equivalence, PMMA is a better choice than polystyrene, as expected and shown for electron dosimetry in earlier studies. Soft‐tissue and water equivalence of different materials is not only a function of depth in the phantom, but also the behavior changes with field size and photon beam energy. Therefore, the soft‐tissue and water equivalence of various phantom materials should be carefully examined before their clinical use. In this regard, MC simulations provide a flexible solution for such analysis where the dose deposition and scatter is correctly accounted for.
